# Building intentions with the theory of planned behaviour: a qualitative assessment of salient beliefs about pharmacy value added services in Malaysia

**DOI:** 10.1111/hex.12416

**Published:** 2015-10-01

**Authors:** Christine Liang Hoay Tan, Mohamed Azmi Hassali, Fahad Saleem, Asrul Akmal Shafie, Hisham Aljadhay, Vincent B. Y. Gan

**Affiliations:** ^1^ Pharmaceutical Services Division (Pharmacy Practice & Development)Negeri Sembilan State Health DepartmentMinistry of Health MalaysiaPenangMalaysia; ^2^ Discipline of Social & Administrative PharmacySchool of Pharmaceutical SciencesUniversiti Sains MalaysiaPenangMalaysia; ^3^ College of PharmacyKing Saud UniversityRiyadhSaudi Arabia; ^4^ Putra Business SchoolUniversiti Putra MalaysiaSerdangSelangorMalaysia

**Keywords:** Malaysia, pharmacy value added services, qualitative, theory of planned behaviour

## Abstract

**Objective:**

To improve pharmaceutical care delivery in Malaysia, the Ministry of Health (MOH) had introduced the concept of value added services (VAS). Despite its reported convenience and advantages, VAS utilization rate is low in the country. The study aims to explore patients’ understanding, beliefs and expectations towards VAS in Malaysia using the theory of planned behaviour (TPB) as the theoretical model.

**Methods:**

A qualitative methodology was used whereby face‐to‐face interviews were conducted with 12 patients who collected partial medicine supplies from government pharmacies. Participants were recruited using purposive and snowball sampling method in the state of Negeri Sembilan, Malaysia. Interviews were audio‐recorded. Verbatim transcription and thematic content analysis were performed on the data.

**Results:**

Thematic content analysis yielded five major themes: (i) attitudes towards using VAS, (ii) subjective norms, (iii) perceived behavioural control, (iv) lack of knowledge and understanding of VAS and (v) expectations towards VAS.

**Conclusion:**

The interviews explored and informed new information about salient beliefs towards pharmacy VAS. The findings suggest that VAS is still in its infancy and a more robust and effective advertising and marketing campaign is needed to boost the adoption rate. Behavioural attitudes, subjective norms and perceived control elements were discussed and serve as important variables of interest in future study. Expectations towards VAS serve as an important guideline to further improve patient‐oriented services.

## Background

In today's competitive and demanding consumer market, subsistence and realization of health‐care institutions depends upon the deliverance of value added services (VAS) to their customers.^1^ In Malaysia, VAS refers to a range of new services optimizing pharmaceutical health‐care delivery to end users by offering additional value, features and convenience to patients when compared to conventional pharmacy counter system.^2^ The concept of VAS was introduced by Ministry of Health (MOH), Malaysia, in 2003 with Integrated Drug Dispensing System (IDDS) being the first ever VAS offered at public health facilities.^2^

In Malaysia, services under the umbrella of VAS include Pharmacy Drive Through (PDT), Postal Medicine (UMP), Pharmacy Appointment Card System (PACS), Integrated Drug Dispensing System (IDDS), SMS and Collect (S&C), Email and Collect (E&C), Telephone and Collect (T&C) and Fax and Collect (F&C). The new delivery service is expected to speed up drug collection time, alleviate queuing trouble, provide creative alternative channels to deliver drugs to the patient's doorstep and ease the stressful parking experience in busy hospitals. At present, VAS is implemented in many government hospitals and primary health clinics. However, not all VAS are available in every health‐care centre. As per the authors’ observation, this can be among few reasons that few Malaysian consumers have adopted VAS so far. Value added services are still a new concept, and many patients are not utilizing these services adequately. Moreover, the perceptions and intentions of Malaysian consumers in adopting VAS are not reported and to the best of our knowledge, there is no available literature on issues pertaining to the acceptance of VAS.

In line to the objectives of this study, a possible measure of intention assessment is attributed to the use of qualitative methods encapsulating models of health behaviours.^3^ Within this context, while the majority of health‐care models focus on perceptions, the theory of planned behaviour (TPB) focuses on the intention to adopt behaviour.^4^ The theory of planned behaviour is an extension of the theory of reasoned action and is underpinned by the assumption that human behaviour is essentially rational and that the immediate antecedent of any behaviour is intention.^5^, ^6^ The TPB adds perceived control over the behaviour, taking into account situations where one may not have complete volitional control over behaviour.^5^ Behavioural intention refers to motivational factors, where stronger intentions to perform the behaviour predict greater likelihood of its performance. Intention indicates how hard people are willing to try or how much of an effort they are planning to exert to perform the behaviour.^7^ According to the TPB, intentions to perform behaviours of different kinds can be predicted with high accuracy from attitudes towards the behaviour, subjective norms (SN) and perceived behavioural control (PBC). These intentions, together with perceptions of behavioural control, account for considerable variance in actual behaviour.^7^ One of the major strengths of this theory is that since its introduction, it has become one of the most frequently cited and influential models for prediction of human social behaviour.^8^, ^9^

Therefore, this study aimed to investigate possible predictors that affect public’ intention to adopt VAS in Malaysia using TPB framework. Questions about salient beliefs of the advantages, approval and motivating/impeding factors of VAS usage were structured to investigate VAS adoption for this study. Other questions to facilitate the qualitative quest were added, focusing on the possible exogenous latent variables that might emerge during the data abstraction process.

## Methods

### Study design, settings and sampling

A qualitative approach was used to gain an in‐depth understanding of perception and intention towards VAS. Malaysian nationals ageing 18 years and above, able to converse in either English or Malay language and had experience collecting their partial medicine supplies from any public pharmacies in the state of Negeri Sembilan were approached for the study. Purposive and snowball sampling technique was used for recruitment.^10^ To target information‐rich and key informants, purposive approach was initially applied to identify three participants from the researcher's social networks with different chronic diseases and collecting different medications.^11^ The participants were later asked to identify other potential participants from their own networks who met the inclusion criteria.

### Study procedure

A semi‐structured interview guide was used for data collection (Table 1). Probing questions were asked in between conversations to clarify the meanings of responses and to gain insight of the topic being discussed. We referred to the following guideline to structure the interview questions.^12^

**Table 1:** Interview guide assessing participant's beliefs towards value added services

Constructs in general	Questions
General questions	What do you understand of value added services (VAS)? How much do you know about VAS? What are your expectations towards VAS?
Attitude	What do you think are the advantages of using VAS? (What are the positive feelings of using VAS?) What do you think are the disadvantages of using VAS? (What are the negative feelings of using VAS)
Subjective Norms	Who do you think would approve or support you if you use VAS? Who do you think would disapprove or discourage you from using VAS?
Perceived Behavioural Control	What are the factors or motivators that make it easy for you to use VAS? What are factors or barriers that make it difficult for you to use VAS?
Demographic (separate sheet)	Name, age, gender, ethnicity, marital status, education and income

1. Positive and negative feelings about using value added services (experiential attitude or affect)
2. Positive and negative attributes or outcomes of using value added services (instrumental attitudes, behavioural beliefs)
3. Individuals or group of people to whom they might listen who are in favour of or opposed to their behaviour to use value added services (normative referents)
4. Situational or environmental facilitators and barriers to use value added services (control beliefs and self‐efficacy)

Experts from Universiti Sains Malaysia validated the interview guide. The guide was piloted with two patients and was modified to improve clarity and length of questions. The preliminary data and conclusion show that the interview questions were sufficient and appropriately phrased to answer research questions and to minimize validity threats.^11^ The respondents, however, expressed that the interview length is cognitively burdensome. Hence, some questions were removed to facilitate participation of interviewees.

### Interview process

All interviewees were briefed about the study before the interviews and debriefed at the end of the session. Interviews were conducted at locations convenient to the participants. All interviews were audio‐recorded and transcribed verbatim by two researchers who were fluent in English and Malay languages. Field notes were used to complement the recording. The duration of interview varied between 30 and 60 min.

### Data abstraction

The first author transcribed the interviews, incorporated field notes and translated conversation from Malay to English. The second author checked all transcriptions against original voice record. Discrepancies were discussed, and a final decision was made after mutual agreement. All participants examined transcripts for precision and accuracy of words, ideas and jargons. Corrections were made accordingly. All participants endorsed signatures on the original manuscripts as proof of authenticity. Data are coded and analysed by the first author and are checked and clarified for data analysis and representation by the second author. Emerging themes served as important variables to help operationalize the constructs of TPB. Exogenous variables apart from TPB were extracted to test for possible variance explained in the next stage survey.

### Ethical approval

The study was approved by Medical Research & Ethics Committee, Ministry of Health, Malaysia (NMRR‐14‐483‐20556). Written consent was obtained from the participants prior to data collection process.

## Results

**Figure 1:**
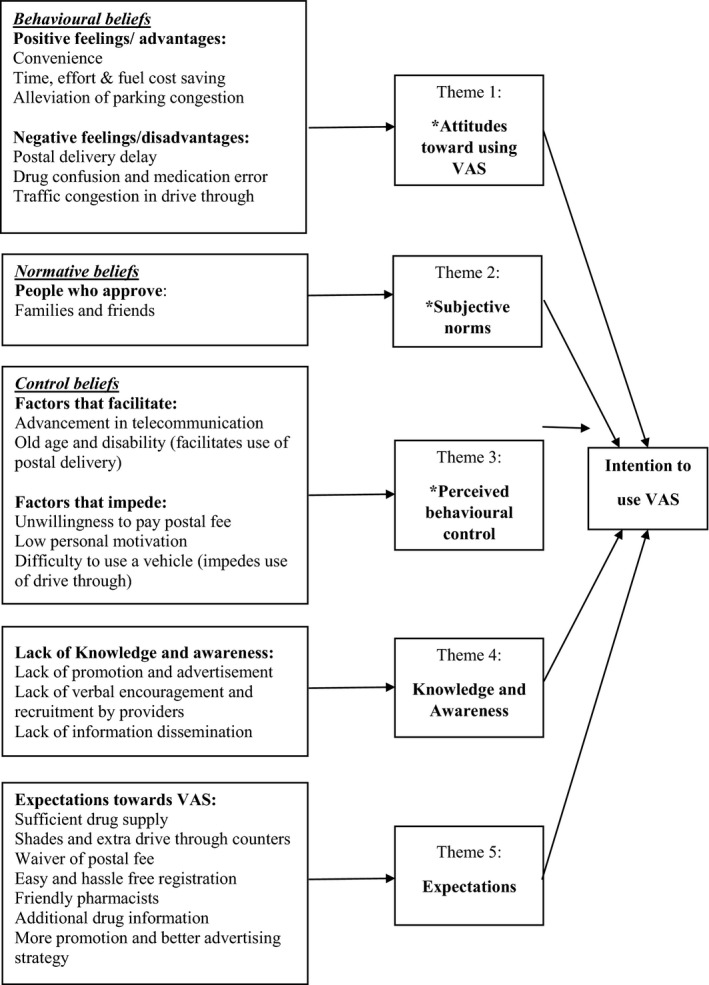
The extended theory of planned behaviour framework to understand patient's intention to adopt pharmacy value added services (Note: Variables with * stand for original predictors from TPB).

Figure 1 displays the TPB theoretical model to understand intention to use VAS. Twelve participants were interviewed. The saturation was achieved at the 11th interview, but the interviews were carried on until 12th to assure the emergence of no new themes. Table 2 illustrates the characteristics of the study participants. During the analysis, five major themes were identified: (i) advantages and disadvantages of using VAS, (ii) subjective norms: approval or disapproval of using VAS, (iii) factors facilitating the use of VAS, (iv) lack of knowledge and awareness among patients and (v) expectation towards VAS in implementation.

**Table 2:** Demographic characteristics of the study participants

Respondent's Label	Age	Gender	Ethnicity	Marital status	Highest education level	Monthly income^a^
AD	43	Female	Malay	Single	Diploma	<RM 2000
DO	64	Female	Chinese	Married	Master's degree	RM 2001–RM 4000
HW	32	Male	Chinese	Married	Undergraduate	RM 4001–RM 6000
MAG	70	Female	Chinese	Married	Higher secondary	Pensioner
MAR	56	Female	Malay	Married	Diploma	RM 2001–RM 4000
MAS	33	Female	Malay	Married	Diploma	RM 2001–RM 4000
MUS	51	Female	Malay	Married	Diploma	RM 2001–RM 4000
GOP	68	Male	Indian	Married	Undergraduate	Pensioner
SF	42	Male	Malay	Married	Undergraduate	>RM 6000
TH	73	Male	Chinese	Married	Higher secondary	Pensioner
VS	69	Male	Indian	Married	Higher secondary	RM 2001–RM 4000
WILL	44	Male	Chinese	Married	Undergraduate	>RM 6000

a RM, Ringgit Malaysia.

### Theme 1: attitudes towards using value added services

#### Positive feelings (advantages)

Respondents were asked to discuss their positive as well as negative feelings towards using pharmacy value added services to refill monthly medication. From the interview, a patient's positive attitudes towards VAS derive from their perceived advantages and benefits of utilizing VAS compared to traditional counter services. Time, effort and fuel cost savings, convenience and alleviation of parking congestion were identified as the most potential advantages that attract them into using VAS.

Ever since they offer this medicine postal delivery service, it really saves a lot of my time and my dad's time; i would say it is a very good service. I think it is very good…excellent…convenient. (SF, 42)

Basically it is creating convenience and also better turn‐around time. My perspective is, it shorten the queuing time in hospital…convenient, time saving, cost saving. (WILL, 44)

Participants were in favour of UMP 1 Malaysia that delivers drug parcels to the patient's home. One participant (MAR, 56) remarked that many geriatrics defaulted their medicines due to difficulties to replenish medicines in hospitals and clinics as most of her children are working and are too busy to assist. She expressed that the UMP service provides a flexible way for the senior citizens to receive their monthly medicine supply and indirectly assist patients to comply with their drug regimen. When asked about the delivery cost, she explained that the small postal fee is irrelevant and trivial compared to the havoc and trouble of returning to the pharmacy on a monthly basis. Many patients who reside in rural areas and villages have difficulties and obstacles in getting transportation to the nearest health‐care centres. The transportation charge by taxis is often much more expensive than the UMP postal fee of RM5–RM10. Senior patients with walking difficulties prefer to stay home most of the time as going out and walking alone in the public might impose higher risk of falling and injury.

#### Negative feelings (disadvantages)

Participants were asked about negative feelings of using VAS, and the feedbacks received were identified as (i) postal delivery delay; (ii) drug confusion and medication error while using postal delivery service; and (iii) traffic congestion at drive through.

##### Postal delivery delay

Past negative experiences with the national postal service weakened the confidence and intention to use UMP. Most participants expressed uncertainty and disappointment in timeliness of service promised. The delay and confusion in using national courier service discouraged many from using UMP to collect medicine. The delivery system could not guarantee the delivery time hence, causing much of insufficiency and resource wastage.

I remember at one time, my dad kept waiting and waiting for the arrival of the medicine …ya, waiting at home, expecting the (drug parcel), he had to stay home whole day… (SF, 42)

I don't like this courier delivery business because they got a habit of coming when you are not home. You know during the last couple of years, I think I can count on the hand, the number of times they come when we were in. Somehow, it does not work for me. So express post becomes delayed post. (VS, 69)

Generally, the quality of postal service generated anxiety and shaken the public's confidence, thus affecting the motivation to use it. Some participants suggested switching the delivery company to increase competency and efficiency.

##### Drug confusion and medication error

Receiving wrong or unexpected medication through UMP is a stressful and fearful experience for some participants. This concern is understandable because patients feel insecure and troubled with unfamiliar or ambiguous drugs with no pharmacist on the spot for consultation. This is especially worrisome in certain pharmacies that constantly switch between generic brands to accommodate low supply of certain brands. Uncertainty and confusion impede the adoption of UMP among patients. They preferred the opportunity to ask a pharmacist at the pharmacy counter during drug collection if they are ambiguous about the medicine received.

We are worried about the medication delivery …the type of medicine deliver to us might not be correct…could be totally different…possible mismatch or mistake. (HW, 32)

##### Traffic congestion at the drive through

Many participants foresee traffic congestions in the near future with the present single drive through lane. Long car queues under sun or rain discourage people from using drive through service. The concern about inefficiency at the drive though counter arises because presently there is only one counter for medicine dispensing. Many participants suggested adding more lanes and counters in the future to facilitate speedy dispensing. Some participants suggested that drive through be restricted to elderly patients. Traffic lights and clear signage to direct the flow of cars at the drive through were also suggested.

People don't like to wait. It is like (burger outlet), sometimes the queue is very long…the kids at the back (keep asking) “when is thing coming out? If the traffic or queue is long, I think it is troublesome. (SF, 42)

I would say that in the long run, when everybody starts to use drive though…there will be jam…I would say that they go according to age group, may be they can give the elderly only. (GOP, 68)

### Theme 2: subjective norms: approval or disapproval of using VAS

#### People who approved using VAS: Families and friends

Family comes first. This was the general expression of many participants. Wives, husbands, children and parents served as important groups of people that affect the participants’ decision with a few stating that friends sometimes affect their decision. Seventy‐three‐year‐old ‘TH’ expressed that he and his wife are living without their children. Both of them will consult each other on daily basis before making any decision. All male participants agreed that their wives are the most important people in their lives. Most female participants also regard their husbands, and sometimes children play very significant roles in their lives. Therefore, we can conclude that the most prominent influential people come from the family and they are expected to mutually agree to a certain degree before making daily decisions.

Well, my wife and I are staying away from my children. We consult each other. (TH, 73)

#### People who disapproved using VAS

A majority of the participants reported that there is no influence of other people upon their disapproval of adopting VAS.

### Theme 3: factors that facilitates or impedes the use of VAS

#### Factors that facilitate VAS adoption

The most salient factors that motivate people to use VAS is the perceived advantages of convenience while collecting medicine, reduction in time spent in facilities, fuel cost savings, saving of annual leave days and an eased vehicle parking experience with drive through and postal delivery.

For me, it is the benefits of these. We can compare to those days. Imagine last time, how difficult it was to take a bus, climb up and down, to the clinic, to collect medicine. No car. When people have cars, people complaint about no parking space in clinic and hospital, complaint about long waiting time. Now this system (VAS) is really very convenient for us, so grab it if it was me. (MAR, 56)

Modernization in telecommunications was also seen to facilitate the adoption of VAS. The provision of hi‐speed data communications and internet connectivity encourages many patients both young and old to use mobile phones to connect to the world. Modern mobile phones support a wide variety of services such as text messaging, MMS, email, internet access, short‐range wireless communication and photography. According to participants, social media such as Facebook has the potential to enhance VAS adoption rate.

Many patients including the old people have modern big hand phone that can access to internet…it is very easy and convenient nowadays, just sms, email, fax or phone, WhatsApp, Facebook, they are good in using them. We have to leave the past, the past is history and taking medicine was very, very hard and troublesome in those old days. Now we have this opportunity with good services, use it. (MAR, 56)

Ageing and disability were seen to be good reasons to use VAS especially postal delivery service. The convenience of not going to clinics to collect medicine attracts senior citizens with mobility difficulties.

When I am older one day, when I can no longer drive, I will use postal service…It saves time, we just need to wait at home, save money, save petrol. (MUS, 51)

#### Factors that impede VAS adoption

Some participants expressed that the postal fee is an obstacle to use UMP. Retired patients who live near the hospital or clinic prefer to personally collect monthly supply because they have plenty of time. Instead of being nagged at home, going to hospital serves a good runaway plan to meet old friends in the canteen for drinks. They do not think that the postal fee is worth paying. Some participant expressed that no postal fee should be charged at any person as the amount is too trivial.

If you ask me what is the reasonable price, I think “free of charge” is the reasonable price. You know why I say that? They are giving me so much of medicine, I am a former government servant, I think the value of the medicine they give to me is more than a hundred dollars. What is the five dollars you want to collect from me for? (VS, 69)

For some, indifference may seem less risky than engagement. Patients’ low motivation to enquire and try new things deflated the efforts of health‐care providers. Many participants reported that even if they knew about VAS, they were not keen to pursue the service because of their laziness.

No one discourage me from using VAS. So far, for me, perhaps it is just…lazy to go and register, that is the only barrier. Laziness I supposed. Nothing else. (MAG, 70)

### Theme 4: lack of knowledge and awareness

Majority of interviewees commented that the promotion and advertisement regarding VAS were lacking and not sufficient to reach to a wider audience. Hence, the lack of knowledge and awareness in patients about the benefits, procedures and type of services strongly affect the ‘take off’ of VAS and adoption outcome. General information of VAS was lacking, and service providers were not seen to recruit more patients into the service programme. The dissemination of information only using conventional posters and banners was questioned and challenged by participants.

I was not aware of it. Nobody explain to me. I did not know about it (VAS)…I was not very aware of the postal service. I was thinking earlier, there must be a cost to it, (since) they post to you, the cost factor. I don't know how much they will be charging, and that is one of the reasons (I did not use it). (GOP, 68)

### Theme 5: expectation towards VAS

Different people expect different outcomes and benefits from VAS. Maintaining sufficient and quality medicine supply is viewed as very important. For drive through pharmacy, large shades is expected to shield against sun and rain during collection and more counters is expected in the near future to alleviate congestion. Postal fee is expected to be waived. Some interviewees suggested government pharmacies to sell medical appliances (glucometer and BP meter) and supplements (vitamins and herbal products) at a lower price within the pharmacy premises which offer VAS.

I feel that the postal service should be free of charges since…it is just a little bit extra, well the government should have (absorbed) the postal fee…especially for those who are poor and needy, I feel that they (government) should pay for them. (MAG, 70)

The standard registration procedures of using VAS must be clearly defined and made easy and hassle free for patients. Some interviewee stressed that systematic unbiased procedures are very important to encourage all ethnic groups to use VAS. It was suggested that VAS registration could be performed via a website and online system. Flexibility in terms of collection time is expected to encourage more people to use VAS.

Hopefully I have the flexibility to go one day before or a day after the given appointment date, something like that…because at our old age, to keep the appointment at that time…is very difficult…you know I missed my hospital appoint‐ment sometimes. (VS, 69)

People expected friendly services. This is true when many participants expressed their preference towards friendly pharmacists. Patients explained that they show more respect and gain confidence in pharmaceutical services if the pharmacists are professional, responsible and be able to handle cases and request in a proper manner and are friendly towards patients. Drug counselling and advice are expected in all services, and good eye contact helped to establish the health‐care provider–client relationship.

I expect friendly pharmacists, and responsible and professional. They also must give us confidence while (we) collect medicine. (MUS, 51)

## Discussion

This qualitative study explored the salient beliefs and opinions from public participants of VAS in the state of Negeri Sembilan, Malaysia.. The most prominent perceived advantages of using VAS reported were time, effort and fuel cost savings arising from the convenience in drug collection and alleviation of parking congestion. The TPB stated that in order to predict a person's intention to perform a behaviour, one needs to know whether the person is in favour of performing the behaviour, how much the person feels the social pressure to do it and whether the person feels in control of the action discussed.^13^ The perceived advantages expressed by interviewees are the positive feelings towards using VAS to collect partial medicine supply. These positive features encourage people to use VAS to collect their medicine. Similarly, the disadvantages of using VAS perceived by interviewees were postal delivery delay, drug confusion and medication error in postal delivery and traffic congestion at the drive through. Lack of confidence in the national courier service is one of the major obstacles to use UMP. Lack of timeliness and punctuality impedes UMP usage among many working people as well as older patients. The redundancy and rigidity of the courier service should be minimized. Perhaps using other courier services might improve the current UMP service since courier services are outsourced.

Medication errors during postal delivery pose a threat to patients’ intention to use UMP. Hence, alternative ways to increase a patient's confidence and to reduce errors are double‐checking of drugs by different pharmacists before delivery and provide patients with a contact number for enquiry purposes. The issues of traffic congestion in single lane drive through can be further analysed and improved by proper and strategic implementation and construction of future drive through counters. Enlargement of the compound dedicated to the drive through lane can be considered if feasible. A drive through building is urged to be built with friendly pick‐up windows and multiple lanes to accommodate more cars simultaneously. In a multiple pick‐up windows setting, suggestion of internal traffic lights and control might serve to mitigate traffic congestion. In future, a detailed and holistic approach in design is important before implementation and constructions of drive through pharmacy outlets.

Generally, when people have negative feelings and evaluations towards a particular behaviour, the ultimate intention to perform the behaviour will be lessened. As intentions are considered the precursors of behaviour, the diminished intentions to use VAS will result in a limited adoption of VAS.^13^ Out study indicates that in order to increase VAS adoption, the advantages and benefits of VAS must be conveyed to patients who have not used VAS. This can be through promotions or any other effective channel of com‐munication relevant to the target audience.

Families, relatives, neighbours and friends are found to be important social factors that influence behavioural intentions in the Malaysian society. Malaysia has a collectivist culture where social norms are valued and individual actions are influenced by people who are important to a person.^14^, ^15^ From this study, the influence of social pressure perceived by an individual to perform or not perform behaviour cannot be denied because all participants agreed resonantly that their families and friends play an important role to approve their decision. We therefore theorize that subjects tend to conform to the norms of the society in making daily decisions. Reference groups, which are the families, neighbours and friends, assert the most social pressure with respect to the behaviour. This hypothesis indirectly suggest that if a patient receives good service and has a positive experience and attitude of using one of the VAS, the possibility of their perspectives being accepted by their family members and friends is high. To attract more customers to the new service, it is important to establish and maintain good relationships between patient and providers. Therefore, we suggest that using effective relationship marketing strategies providers can market VAS to other new patients using existing patients who value VAS positively.

The TPB model postulates that perceived control reflects people's confidence and capability to perform the target behaviour.^13^ The advancement in telecommunication and modern gadgets were seen to motivate certain groups of users in using SMS, email or fax to collect medicines. Social media platforms like Facebook, Twitter and WhatsApp can be used to improve public's awareness and promotion of VAS. This technology factor has the potential to increase the VAS adoption rate among Malaysians. This finding gives us some preliminary encouragement to suggest to providers and policymakers to adopt modern communication tools in future health promotion strategies. Value added services messages and advertisements can be conveyed to target users using both advanced and traditional health promotion methods.

While VAS offers positive attributes, some drawbacks and system weaknesses withheld patients from using VAS. Unforeseen factors beyond administrative control seem to create inertia in new patients to adopt VAS and hence retard the growth and progress of VAS among Malaysian public. The study found that the patient's unwillingness to pay the postal fee while using the postal delivery service would reduce the adoption rate. A majority of patients suggested waiving the postal fee because the value of the postal fee is not justifiable to the amount of free drugs given to patients. It is also reasoned that senior citizens who are more willing to use postal delivery are retired people with no income; therefore, free delivery service is expected. Low personal motivation and difficulty to use a vehicle were also found to impede VAS adoption. While old age encourages postal delivery usage, physical disability to operate a vehicle prevents the adoption of drive through service. This finding suggests that drive through promotions can be targeted to the younger generation who are in possession and can drive a vehicle.

The interviews found that a large number of patients still do not know the existence and the benefits of using VAS to collect medicine. Lack of awareness and knowledge about the advantages and implementation of VAS impede intention to use. Thus, policymakers and providers are urged to look into the matter to find ways of reaching out to the targeted population. We strongly believed that this lack of knowledge about VAS deters consumers because they feel ambiguous and lacking in confidence about the new systems. From the study, patients suggested that the lack of verbal explanation and encouragement from pharmacists might result in low recruitment and adoption rates. New users view lack of VAS information dissemination from providers to users to threaten adoption. The fact that many respondents did not know about the existence of VAS suggest that current promotion and communication methods fail to reach the expected population. Our experience while conversing with participants suggested that a more strategic and creative advertising method should replace the traditional ones. It is therefore suggested to revise the recruitment methods and promotion strategies. The future selection of location for drive through construction should take into account the availability of public transport facilities such as a nearby bus stop and cab station. This is so that poor patients with no personal vehicle can walk to the drive through window without much physical exacerbation.

We found that participants had various expectations towards VAS including sufficient drug supply, clear standard operating procedures, flexibility in drug collection schedules and professionally friendly pharmacists. Medication counselling and advice, clear drug labels and instructions, and additional information about health care‐related products are expected from pharmacists in daily VAS dispensing. Expectation might serve as antecedent to intention to use VAS or vice versa. Further study is required to investigate how expectations affect intention.

## Conclusion

The interviews explored and informed new information about salient beliefs towards pharmacy VAS in Negeri Sembilan, Malaysia. The findings suggest that VAS is still in its infancy, and a more robust and effective advertising and marketing campaign is needed to boost the adoption rate. Behavioural attitudes, subjective norms and perceived control elements were discussed and serve as important variables of interest in future study. Expectations towards VAS serve as an important guideline to further improve patient‐oriented services.

## Limitation

As an exploratory study, the study aimed to identify salient beliefs to inform the development of future quantitative research. It is understood that some element of bias exist in self‐report data; however, serving as exploratory research, this study was identified as the most effective way to collect preliminary information. Furthermore, predictors of intention do not always determine behavioural change, and reconfirmation through a large‐scale study is needed, which is also a potential limitation to this study.

## Declaration of conflicting interests

The authors declared no potential conflicts of interest with respect to the research, authorship and/or publication of this article. The usual disclaimer applies. No funding was received for this study.
